# The downregulation of Mcl-1 via USP9X inhibition sensitizes solid tumors to Bcl-xl inhibition

**DOI:** 10.1186/1471-2407-12-541

**Published:** 2012-11-21

**Authors:** Chander Peddaboina, Daniel Jupiter, Steven Fletcher, Jeremy L Yap, Arun Rai, Richard P Tobin, Weihua Jiang, Philip Rascoe, M Karen Newell Rogers, W Roy Smythe, Xiaobo Cao

**Affiliations:** 1 Department of Surgery, Scott & White Memorial Hospital and Clinic, The Texas A&M University System, Health Science Center, College of Medicine, Temple, TX 76504, USA; 2 Department of Pharmaceutical Sciences, University of Maryland School of Pharmacy, Baltimore, MD 21201, USA; 3 Boston University School of Medicine, 72 East Concord Street, Boston, MA 02118, USA

**Keywords:** Mcl-1, Bcl-xL, USP9X, Ubiquitination, Cancer

## Abstract

**Background:**

It has been shown in many solid tumors that the overexpression of the pro-survival Bcl-2 family members Bcl-xL and Mcl-1 confers resistance to a variety of chemotherapeutic agents. Mcl-1 is a critical survival protein in a variety of cell lineages and is critically regulated via ubiquitination.

**Methods:**

The Mcl-1, Bcl-xL and USP9X expression patterns in human lung and colon adenocarcinomas were evaluated via immunohistochemistry. Interaction between USP9X and Mcl-1 was demonstrated by immunoprecipitation-western blotting. The protein expression profiles of Mcl-1, Bcl-xL and USP9X in multiple cancer cell lines were determined by western blotting. Annexin-V staining and cleaved PARP western blotting were used to assay for apoptosis. The cellular toxicities after various treatments were measured via the XTT assay.

**Results:**

In our current analysis of colon and lung cancer samples, we demonstrate that Mcl-1 and Bcl-xL are overexpressed and also co-exist in many tumors and that the expression levels of both genes correlate with the clinical staging. The downregulation of Mcl-1 or Bcl-xL via RNAi was found to increase the sensitivity of the tumor cells to chemotherapy. Furthermore, our analyses revealed that USP9X expression correlates with that of Mcl-1 in human cancer tissue samples. We additionally found that the USP9X inhibitor WP1130 promotes Mcl-1 degradation and increases tumor cell sensitivity to chemotherapies. Moreover, the combination of WP1130 and ABT-737, a well-documented Bcl-xL inhibitor, demonstrated a chemotherapeutic synergy and promoted apoptosis in different tumor cells.

**Conclusion:**

Mcl-1, Bcl-xL and USP9X overexpression are tumor survival mechanisms protective against chemotherapy. USP9X inhibition increases tumor cell sensitivity to various chemotherapeutic agents including Bcl-2/Bcl-xL inhibitors.

## Background

Despite improvements in the accuracy of clinical staging for solid cancers, the survival rates for patients affected with these tumor types have improved only modestly over the last few decades. Many solid tumors are unresponsive to conventional therapy due to the resistance of the tumor cells to programmed cell death. The downregulation of Bcl-xL has been shown to induce apoptosis and increase chemosensitivity [1,2] but resistance to chemotherapy is still observed in some cancer cells even after Bcl-2/Bcl-xL inhibition [3,4]. Recent reports have revealed that the overexpression of Mcl-1 compensates for the loss of the anti-apoptotic function of Bcl-2/xL [5,6]. A reduction in Mcl-1 significantly enhances the sensitivity of cancer cells to ABT-737 and other chemotherapeutics [7-9]. In addition, the forced overexpression of Mcl-1 in transgenic mice leads to a significantly increased incidence of B-cell lymphoma [10]. Hence, the cumulative evidence to date suggests that Mcl-1 overexpression may function as an additional survival mechanism that protects cancer cells against conventional therapies.

Mcl-1 expression, just like Bcl-xL expression, is highly induced under conditions that are conducive to survival and by differentiation signals from cytokines and growth factors [11,12]. Mitogen-activated protein kinase (MAPK)-phosphatidylinositol-3 (PI3K)- and Janus kinase (JAK)/signal transducer and activator of transcription (STAT)-dependent pathways have all been implicated in the stimulation of Mcl-1 transcription, acting via specific transcription factor response elements in the Mcl-1 gene promoter [13-15]. However, the direct phosphorylation of Mcl-1 also plays an important role in controlling its expression and function. Mcl-1 can be phosphorylated in its PEST region, and thus stabilized, upon ERK activation [16].

Additionally, Mcl-1 is regulated by a subtle balance between ubiquitination and deubiquitination. Two E3 ligases have been implicated in Mcl-1 turnover. The first of these is Mcl-1-ubiquitinating ligase E3 (MULE) which possesses a BH3 domain similar to that of proapoptotic BAK that allows it to target Mcl-1 [17]. Interestingly, although the RNAi-mediated silencing of MULE slows the Mcl-1 turnover rate, degradation of this protein nevertheless still occurs, suggesting that additional pathways can promote Mcl-1 elimination [18]. The second E3 ligase, SCFβ-TrCP, was discovered to only recognize Mcl-1 that has been phosphorylated by GSK3 at Ser159 [19]. This interaction between SCFβ-TrCP and Mcl-1 is facilitated by phosphorylation of the same serine and threonine residues that have been identified previously as potential sites of recognition by the X-linked ubiquitin specific peptidase 9 (USP9X), a deubiquitinase (DUB) [20]. Hence, it is possible that SCFβ-TrCP and USP9X compete for Mcl-1 binding. USP9X binds Mcl-1 protein and removes the Lys 48-linked polyubiquitin chains that normally mark it for proteasomal degradation. Mcl-1 ubiquitination is thus offset by the activities of USP9X and it has been reported that increased USP9X expression correlates with increased Mcl-1 protein levels and a poor prognosis in lymphoma patients [20]. The silencing of USP9X using siRNAs increases the sensitivity of CML cells, to imatinib and other apoptotic stimuli [21]. The deubiquitination activities of USP9X can be inhibited by WP1130, a partially selective DUB inhibitor [22]. It has been demonstrated in this regard that a reduction in the Mcl-1 levels in WP1130-treated cancer cells parallels the inhibition of USP9X activity.

In our current study, we further tested the hypothesis that Mcl-1 and Bcl-xL are both overexpressed in colon and lung cancers. Our analysis reveals that the overexpression of both of these anti-apoptotic proteins causes resistance to chemotherapeutic agents. In addition, the blocking of USP9X activities using a small-molecule inhibitor decreases Mcl-1 expression by promoting its degradation and thus sensitizes tumor cells to chemotherapeutic agents.

## Methods

### Cell culture

I45, REN (human mesothelioma cell lines), A549, H1299 and H23 (lung cancer cell lines) as well as DLD-1 and HCT116 (colon cancer cell lines) were purchased from the American Type Culture Collection (Manassas, VA). DLD-1, H1299, H23, I45 and REN were cultured in 10% fetal bovine serum (FBS)-supplemented RPMI 1640 medium. A549 cells were cultured in 10% FBS-supplemented F12 medium. HCT-116 cells were cultured in McCoy’s 5A medium containing 10% fetal bovine serum. Authentication of these cell lines was performed by the ATCC.

### Reagents

Cycloheximide, 5-FU, Taxol, PS341, WP1130 and ABT-737 were obtained from Selleck Chemicals (Houston, TX). The HDAC inhibitor SAHA (suberoylanilide hydroxamic acid) was purchased from Biovision (Mountain View, CA). The rabbit anti-human USP9X polyclonal antibody used was obtained from Bethyl Laboratories (Montgomery, TX). Rabbit antibodies against Bcl-xL and Mcl-1 were purchased from Santa Cruz Biotechnology Inc. (Santa Cruz, CA). Mouse monoclonal anti β-actin was obtained from Sigma (Saint Louis, MO). The siRNA transfection reagents, and siRNAs targeting Bcl-xL, Mcl-1 and a control scrambled siRNA, were obtained from Ambion Biotechnology, Inc. (Austin, TX).

### Apoptosis assay

After various treatments, cancer cells were stained for Annexin-V using a FITC–Annexin-V staining kit (Invitrogen, Carlsbad, CA) and then measured with BD FACSCanto II Flow cytometry. Flow cytometry data were analyzed using FlowJo software (Tree Star Corp, Ashland, OR).

### Cell proliferation assays

The effects of various inhibitors on cell viability were assessed in quadruplicate samples using the 2,3-bis(2-methoxy-4-nitro-5-sulfophenly)-5-[(phenylamino) carbonyl]-2H-tetrazolium hydroxide (XTT) assay (Trevigen, Inc. Gaithersburg, MD). Cancer cells were seeded and incubated in 96-well, flat-bottomed plates in 10% FBS-supplemented culture medium 24 hours before drug treatment. The cells were then exposed to various inhibitors at the indicated concentrations at 37°C in 5% CO_2_ for 72 hours. The medium was removed and replaced with 150 μl fresh medium containing XTT, and the cells were further cultured in the CO_2_ incubator at 37°C for 5 hours. Absorbance was determined on a plate reader at 492 nm.

### Western blotting analysis

Cancer cells were lysed using urea containing lysis buffer and equal amounts of total proteins were resolved on 4–20% Tris-glycine gels and transferred onto a nitrocellulose membrane. The membranes were then co-incubated with a rabbit anti-human Bcl-xL polyclonal antibody, a rabbit anti-human Mcl-1 monoclonal antibody, rabbit anti-human USP9X polyclonal antibody and a mouse anti-human β-actin antibody overnight. Antibody binding was then detected using chemiluminescence (Cell Signaling Technology, Danvers, MA) and signals were visualized by autoradiography.

### Clinical tumor specimens and immunohistochemistry

Formalin-fixed, paraffin-embedded tissue from colon adenocarcinoma and lung adenocarcinoma were examined for expression levels of Mcl-1, Bcl-xL and USP9X protein. All samples were histologically confirmed and patient identities were removed. These tumor tissue slides were deparaffinized in xylene, subjected to antigen retrieval, and following endogenous peroxidase quenching were blocked in horse serum and incubated overnight with a rabbit anti-human Bcl-xL polyclonal antibody, rabbit anti-human Mcl-1 monoclonal antibody, or rabbit anti-human USP9X polyclonal antibody. The slides were then incubated with a biotinylated goat secondary anti-rabbit antibody (Vector Laboratories, Burlingame, CA) for 30 minutes and the resulting signals were detected using streptavidin-biotin-peroxidase complex (Vector Laboratories) and diaminobenzidine (DAB) (Vector Laboratories). The slides were counterstained with hematoxylin (Sigma-Aldrich, St. Louis, MO) and the images were captured with a digital camera. The signals were then measured using ImageScope Software (Aperio Technologies Vista, CA). Positivity was quantified by specifying a hue value of 0.1 and hue width of 0.33 for a standardized area of the tumor tissue.

Approval for this study was obtained from the Institutional Ethics Review Board at the Scott & White Memorial Hospital and Texas A &M Health Science Center. The study was conducted in compliance with the Helsinki Declaration.

### Statistics

The co-existence of Mcl-1 and either Bcl-xL or USP9X expression in tumor cells were assessed using the Chi-square and Fisher exact tests. A Pearson correlation between the Bcl-xL, USP9X and Mcl-1 expression profiles was calculated using R statistical software ( http://www.r-project.org). The association of protein expression and clinical staging of the tumor samples was determined using linear regression.

## Results

### Mcl-1 and Bcl-xL are co-overexpressed in multiple solid tumor types

**Figure 1:**
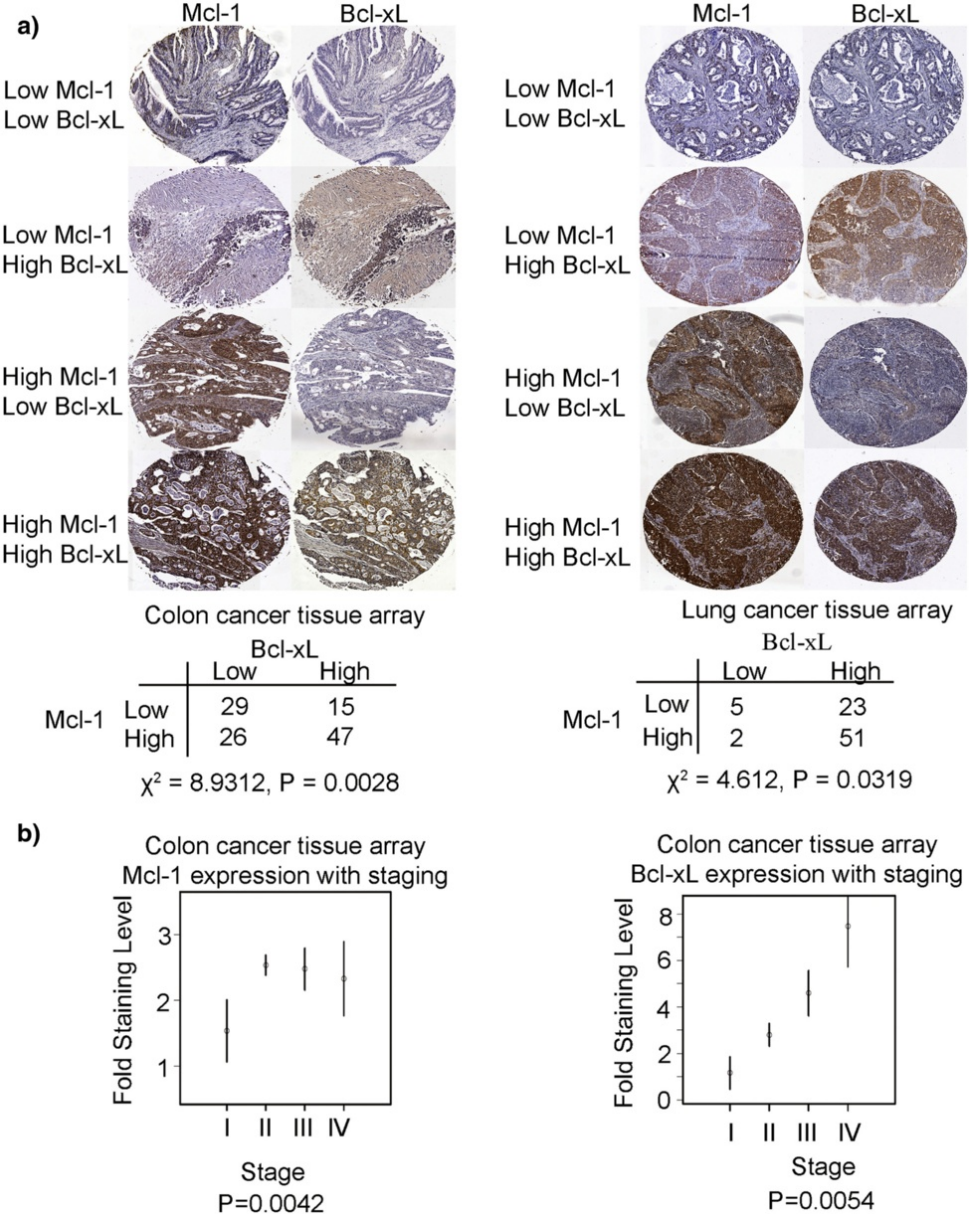
**Mcl-1 and Bcl-xL are co-overexpressed in multiple solid tumor types.** (**a**) Mcl-1 and Bcl-xL expression patterns in lung and colon adenocarcinomas. The co-existence of Mcl-1 and Bcl-xL expression in tumor cells on the tissue slides was assessed using the Fisher exact and Chi-square tests. (**b**) The association between Bcl-xL or Mcl-1 expression and clinical staging in colon cancer samples was measured using linear regression. Protein expression levels between stages were compared using pre-planned contrasts. The mean levels within each stage, plus or minus one standard error, are plotted.

To evaluate the correlation between Bcl-xL and Mcl-1 expression in lung and colon cancer, we analyzed human non-small cell lung adenocarcinoma and colon adenocarcinoma samples by immunohistochemistry using antibodies against these two proteins. As shown in Figure 1a, there were strong associations observed between the expression of Mcl-1 and that of Bcl-xL in both the lung and colon cancer samples. In the 117 human colon cancer samples we analyzed, 47 specimens stained positively for both proteins and a further 29 samples showed weak co-staining for both factors (Chi-squared test, P=0.0028; Fisher test, P=0.0021). In the 81 lung cancer samples tested in this analysis, 51 samples showed strong positive staining for both proteins and five samples showed co-staining at low levels (Fisher exact test, P=0.045; Chi-square value of 4.612, P=0.0319). There were further relationships observed between Mcl-1 and Bcl-xL protein expression and tumor staging in colon cancer samples (Figure 1b). Mcl-1 expression was found to increase with the staging grade (P=0.0042), (P=0.0036 from I to II; P=0.016 from I to III; and P=0.090 from I to IV). Bcl-xL expression was also found to be significantly associated with staging (P=0.0054), with stage I lesions showing significantly different levels of this protein compared with stage III (P=0.033) and stage IV (P = 0.004) tumors. Tumor staging data were not available for the lung cancer samples.

### Tumor cells expressing high levels of Mcl-1 and Bcl-xL protein exhibit chemoresistance

**Figure 2:**
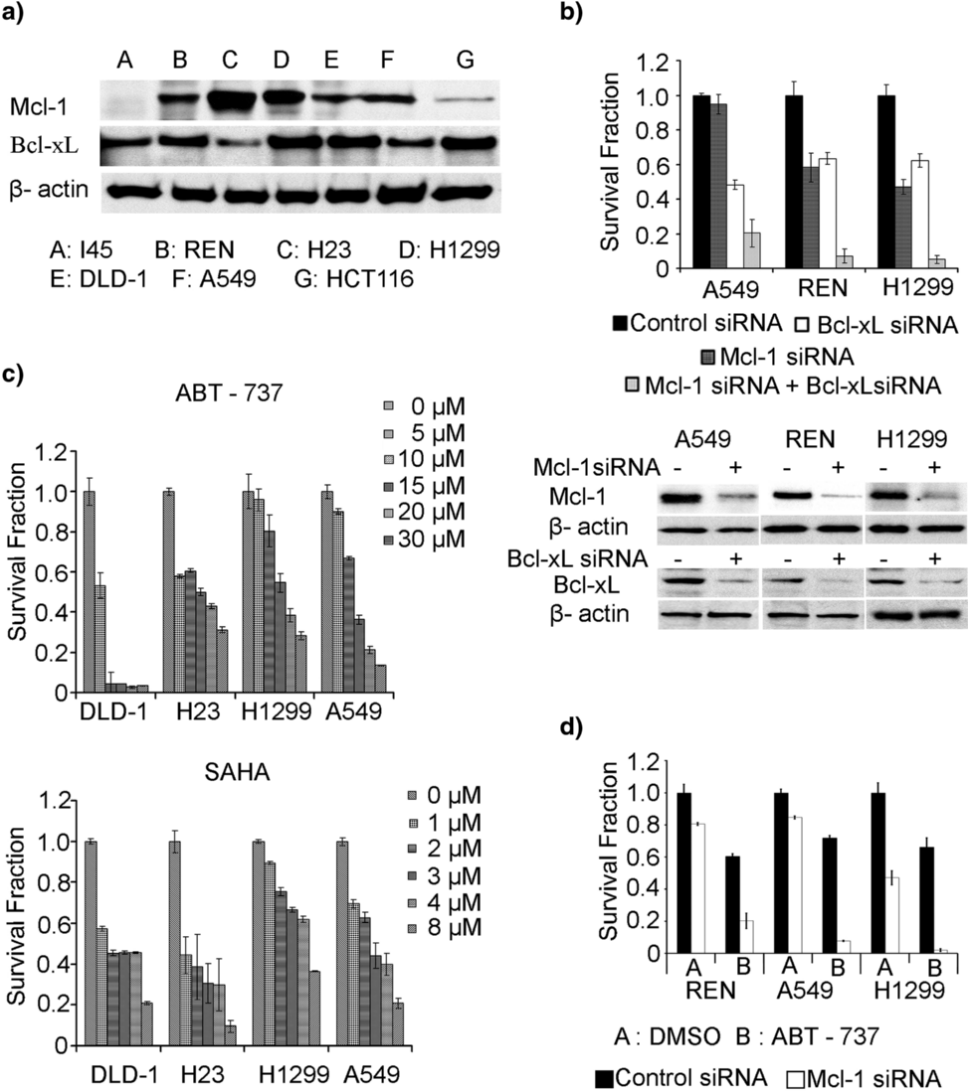
**Tumor cells expressing high levels of Mcl-1 and Bcl-xL protein exhibit chemoresistance.** (**a**) Basal expression of Mcl-1 and Bcl-xL in various lung, colon and mesothelioma cancer cell lines determined by western blot analysis. (**b**) Mcl-1 and Bcl-xL contribute to cancer cell survival. A549, REN and H1299 cells were transfected with control siRNA, Mcl-1 siRNA, Bcl-xL siRNA, or a combination of both, via electroporation using Nucleofector II from Lonza (Switzerland). The cells were then seeded in 96 well plates, and cultured for 72 h. An XTT assay was performed following the manufacturer’s protocol to evaluate cell viability. Western blotting was used to measure the Bcl-xL and Mcl-1 protein levels after specific siRNA transfection. (**c**) To determine the chemo-response of DLD-1, H23, H1299, and A549 cells to SAHA and ABT-737, the cells were plated at a density of 5000 per well and treated with different concentrations of these chemotherapeutic agents over a 72 h incubation. An XTT assay was then performed in quadruplicate for each treatment condition and the experiments were repeated three times. (**d**) Mcl-1 was knocked down by siRNA in A549, REN and H1299 cells which were plated in a 96 well format. A control siRNA experiment was included. The cells were then treated with DMSO (control) or ABT-737 (IC30 doses) for 72 h, followed by an XTT assay for cell viability. The results were averaged from quadruplicate analyses for each treatment condition and the experiments were repeated three times.

To test the hypothesis that high Mcl-1 and Bcl-xL expression contributes to drug resistance, including resistance to Bcl-xL inhibitors, the baseline protein expressions of Bcl-xL and Mcl-1 in multiple cell lines were examined via western blotting (Figure 2a). The results demonstrated the concurrent expression of both Mcl-1 and Bcl-xL in most cell lines, corroborating the immunostaining results in both lung and colon tumor tissues shown in Figure 1. To evaluate the role of Mcl-1 and Bcl-xL in tumor cell survival, knockdowns of each factor alone and in combination were performed with small interfering RNAs (siRNAs) in A549, REN and H1299 cell lines that overexpress both Mcl-1 and Bcl-xL proteins. Unilateral Mcl-1 reduction caused cell death at 10%, 45% and 50% levels in A549, REN and H1299 cells, respectively, whilst a Bcl-xL knockdown alone caused 50%, 37% and 40% rates of cell death in these cells. However, the co-inhibition of both proteins by RNAi resulted in low cell survival with an almost 80-90% drop in viability (Figure 2b). Bcl-xl and Mcl-1 reductions via siRNAs were demonstrated using western blotting (Figure 2b).

To examine whether Mcl-1 contributes to Bcl-xL inhibitor resistance, we next evaluated the viability of various cell lines with different Bcl-xL and Mcl-1 expression profiles in the presence of ABT-737 (Figure 2c). The colon adenocarcinoma cell line DLD-1, which expresses relatively lower Mcl-1 levels, but high Bcl-xL expression, was found to be sensitive to Bcl-xL inhibition via ABT-737. A549 and H1299 cells, which express relatively high levels of Bcl-xL and Mcl-1, and H23 cells, which shows strong Mcl-1 expression and low Bcl-xL expression, all demonstrated resistance to ABT-737. Similar levels of resistance to SAHA, a histone deacetylase inhibitor, were only observed in those cell lines with both Bcl-xL and Mcl-1 overexpressions. To further assess the role of Mcl-1 in the resistance to Bcl-xL inhibition, A549, H1299 and REN cells were transfected with control siRNAs or Mcl-1 siRNAs and then exposed to ABT-737 at their calculated IC30 doses (10, 12 and 20 μM, respectively). After Mcl-1 reduction and ABT-737 treatment, survival fractions of A549, H1299, and REN cells were decreased to 10%, 5% and 19% respectively, while in control siRNA transfected and ABT-737 treated cells showed 70%-75% viabilities (Figure 2d). This data indicate that reduced Mcl-1 expression enhances the sensitization of cells to Bcl-xl inhibition.

### Mcl-1 and USP9X are both overexpressed in colon and lung cancers

**Figure 3:**
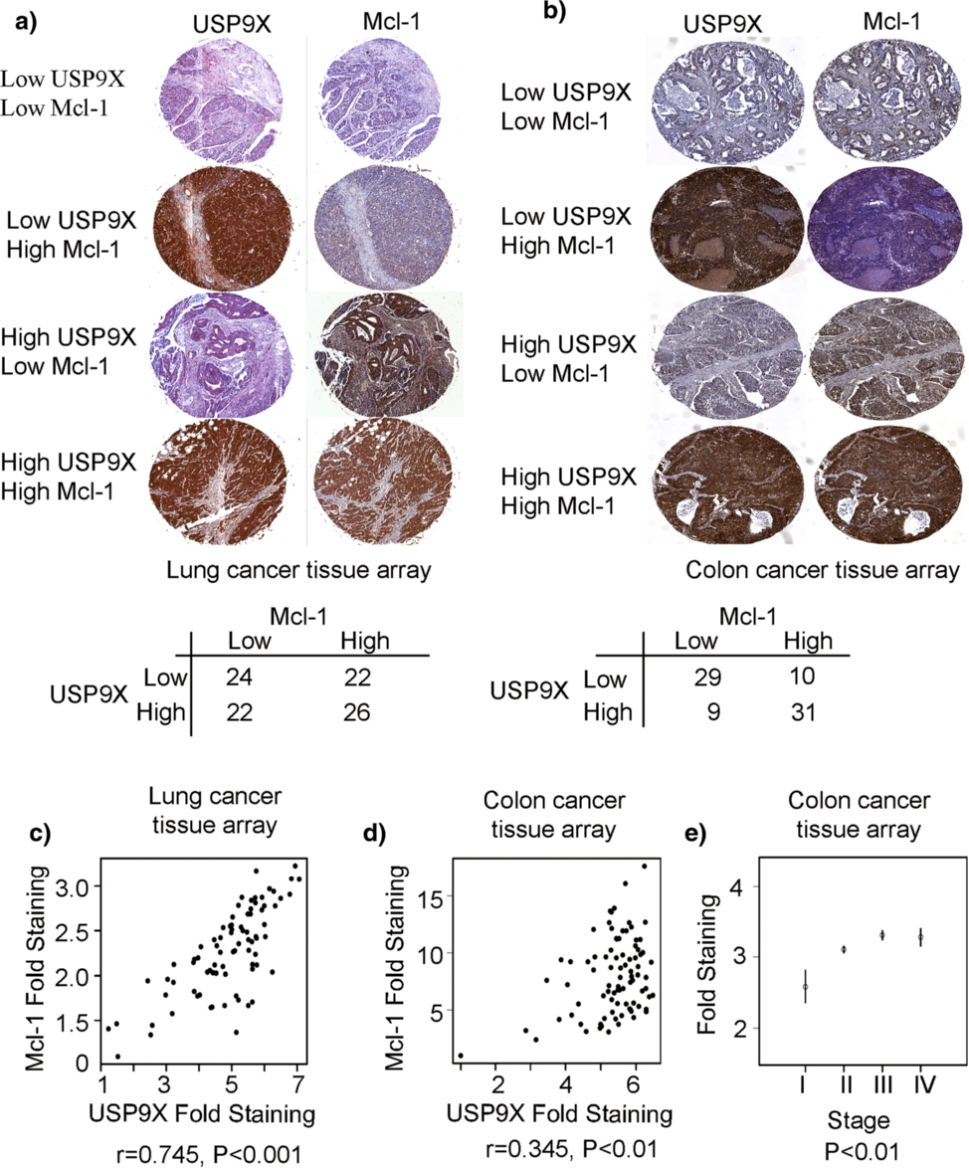
**Mcl-1 and USP9X are co-overexpressed in colon and lung cancers.** (**a**) Lung adenocarcinoma tissue slides were stained for USP9X and Mcl-1 proteins. (**b**) On slides containing colon adenocarcinoma tissue samples, USP9X and Mcl-1 staining was performed. (**c**) Pearson correlation between the USP9X and Mcl-1 expression data for 94 lung adenocarcinoma samples was calculated using R statistical software. (**d**) Pearson correlation between USP9X and Mcl-1 expression in 79 colon adenocarcinoma samples was determined using R statistical software. (**e**) The correlation between USP9X expressions and the clinical staging of different colon cancer samples was determined using linear regression. The mean levels within each stage, plus or minus one standard error, were plotted.

USP9X was recently identified as an Mcl-1 deubiquitinase [20]. To further elucidate the relationship between USP9X and Mcl-1 in clinical samples, the protein expression levels of these factors were evaluated in a panel of 94 human non-small cell lung adenocarcinoma specimens by immunohistochemistry (Figure 3a). The results demonstrated a strong correlation between USP9X and Mcl-1 expression levels (r=0.745, P<0.001; Figure 3c). We performed the same analyses in a series of 79 colon tumor samples (Figure 3b) and observed a moderate correlation between the expression of USP9X and Mcl-1 (r=0.345, P<0.001; Figure 3d). In terms of a linear model for the expression of USP9X in colon carcinoma, this was found to be significant (P=0.00083). In terms of tumor staging, we found that stages I-II (P=0.00043), I-III (P<0.001) and I-IV (P=0.0011) were significantly different. In each case the higher stage showed higher expression values. The difference between stages II and III (P=0.032) was also found to be significant, with stage III tissues showing higher expression of USP9X (Figure 3e).

### USP9X activity regulates Mcl-1 expression

**Figure 4:**
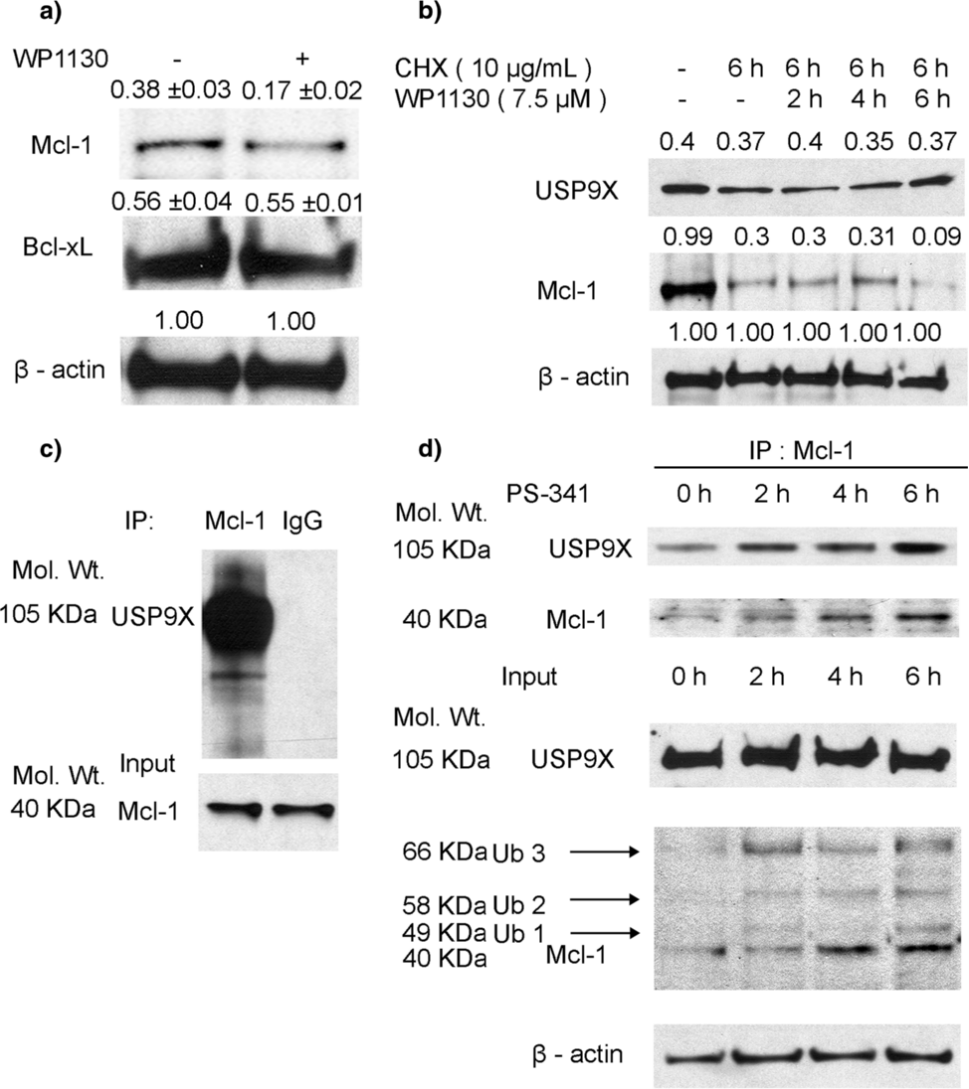
**USP9X activity regulates Mcl-1 expression.** (**a**) H1299 cells were treated with 10 μM WP1130 for 6 h and the subsequent downregulation of Mcl-1 was confirmed by western blotting. No change was observed in the Bcl-xL protein levels. The β-Actin expression levels were detected to normalize for protein loading. Proteins were quantified using the UN-SCAN-IT automatic digitizing system. Those intensities of the signals of Bcl-xl and Mcl-1 are relative to Actin. The mean ± SD represented the triplicate of three repeated western blotting. (**b**) A549 cells were plated in 10 cm tissue culture dishes and upon reaching 70% confluency were treated with 10 μg/ml CHX for 6 h or exposed to a combination of 7.5 μM WP1130 for 2, 4 and 6 h and 10 μg/ml CHX for 6 h. Mcl-1 and USP9X signals were then determined by western blotting. Proteins were quantified using UN-SCAN-IT. Those intensities of the signals of USP-9X and Mcl-1 are relative to Actin. (**c**) Mcl-1 proteins were immunoprecipitated from A549 cell lysates using anti-Mcl-1 antibody or control IgG. USP9X signals were then detected by western blotting. (**d**) A549 cells were exposed to a proteasomal inhibitor PS341 for 2, 4 and 6 h and the cells were then harvested and lysed. Mcl-1 was immunoprecipitated from these lysates with an anti-Mcl-1 antibody. USP9X signals and Mcl-1 signals were then detected by western blotting. Ub1 represents mono-ubiquitylated Mcl-1. Ub2 represents ubiquitylated Mcl-1 conjugated with two ubiquitins. Ub3 represents ubiquitylated Mcl-1 conjugated with three ubiquitins.

To explore the role of USP9X inhibition in Mcl-1 expression regulation, H1299 cells were exposed to the USP9X inhibitor WP1130 [23] for six hours and Mcl-1 expression was subsequently examined via western blotting. As shown in Figure 4a, exposure to WP1130 led to a 50% reduction of Mcl-1 expression in these cells, whereas the Bcl-xL expression levels remained unchanged. To obtain additional evidence that USP9X protects Mcl-1 from degradation, A549 cells were exposed to the protein synthesis inhibitor cycloheximide (CHX) alone or in combination with WP1130. The CHX and WP1130 combination at six hours caused a significantly higher reduction of Mcl-1 than CHX alone. This result indicates that the inhibition of USP9X accelerates Mcl-1 degradation (Figure 4b) and hence that USP9X activities are critical for Mcl-1 stability. Immunoprecipitation (IP) western blotting was employed to further explore the physical interaction between USP9X and Mcl-1 in cancer cells and a strong direct association was observed (Figure 4c). To further probe the role of USP9X in preventing Mcl-1 degradation, A549 lung cancer cells were exposed to the proteasomal inhibitor PS-341. Increased binding between USP9X and Mcl-1 (Figure 4d) was detected by IP western blot, whilst Mcl-1 expression was found to be elevated by PS341. PS-341 induced Mcl-1 ubiquitylations were demonstrated in Additional file 1: Figure S1. These findings confirmed that USP9X is an Mcl-1 deubiquitinase and thereby regulates Mcl-1 degradation.

### USP9X inhibition sensitizes tumor cells to various chemotherapies

**Figure 5:**
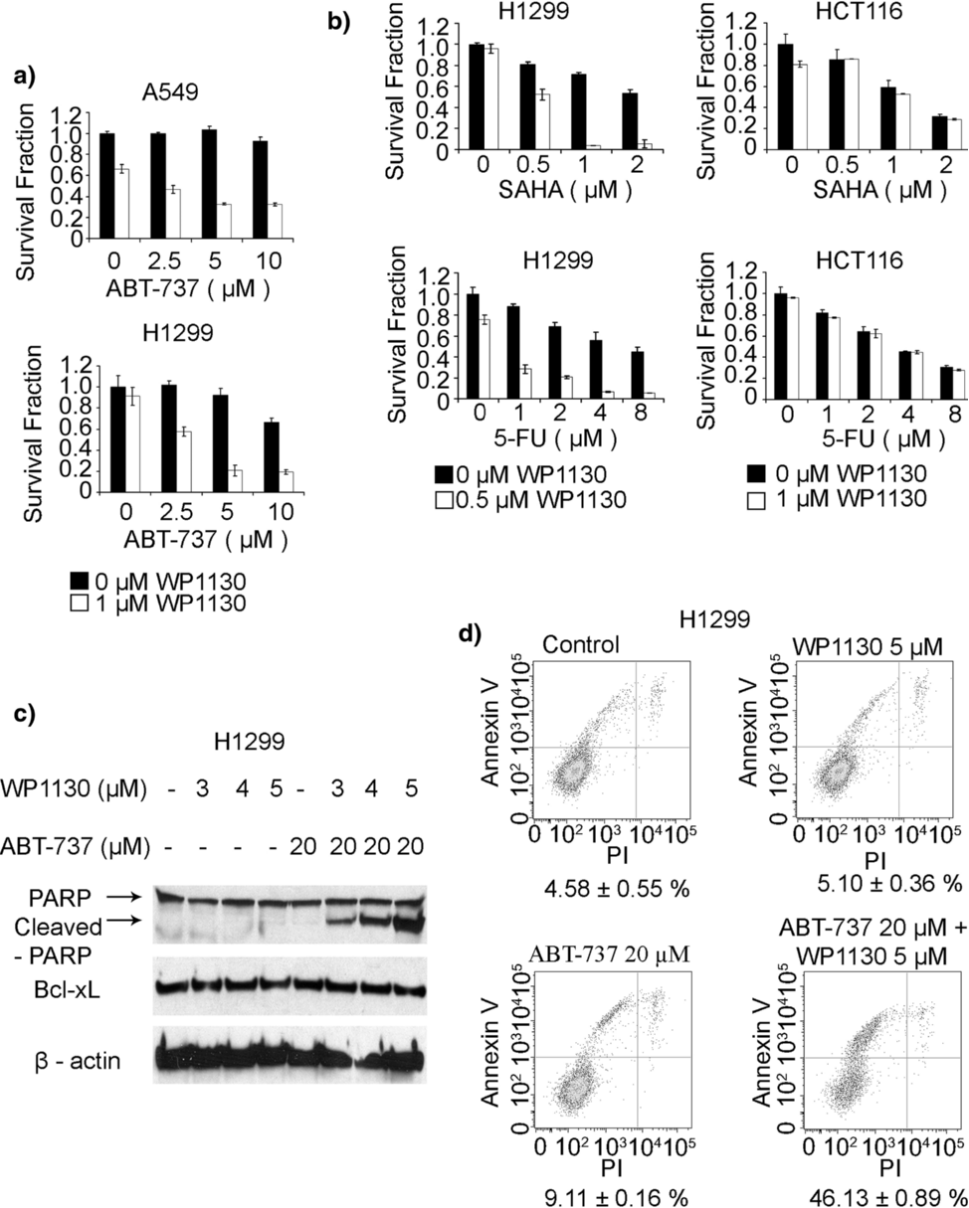
**USP9X inhibition sensitizes tumor cells to various chemotherapies.** (**a**) A549 and H1299 cells were seeded into 96 well plates (5000 cells per well) and then exposed to various combinations of inhibitors including 1 μM WP1130 and different doses of ABT-737. An XTT assay was performed 72 h after treatment. The data shown are the average of triplicate measurements for each treatment condition. Experiments were repeated three times. (**b**) H1299 and HCT-116 cells were seeded into 96 well plates and then exposed to various combinations of WP1130 and either SAHA or 5-FU. An XTT assay was performed 72 h after treatment. The data shown are the average of triplicate measurements for each treatment condition. Experiments were also repeated three times. (**c**) WP1130 enhances ABT-737 induced apoptosis. H1299 cells were treated with different concentrations of WP1130 (0, 3, 4 or 5 μM) in combination with ABT-737 (20 μM) over a 24 h period. The PARP cleavage profile in these samples was then documented via western blotting using an anti-PARP antibody. β-actin was detected as a loading control. (**d**) The apoptotic cell population following various WP1130 and ABT-737 treatments was stained with Annexin-V and quantified using flow cytometry. The data shown are the average of quadruplicate assessments for each condition. Experiments were repeated twice.

To explore the therapeutic potential of USP9X inhibition in conjunction with various chemotherapeutics, we evaluated the capacity of WP1130 in combination with ABT-737 to increase the chemosensitivity of H1299 and A549 cell lines. With concurrent WP1130 treatment in A549 and H1299 cells, the cytotoxic response to ABT-737 increased drastically (Figure 5a). Furthermore, WP1130 was found to sensitize the H1299 cell line (High Mcl-1 expression), but not the HCT116 cell line (low Mcl-1 expression), to SAHA and 5-FU treatments (Figure 5b). Similar sensitization outcomes were observed in multiple cancer cell lines such as REN, DLD-1 and LOVO. Western blot analysis of H1299 further revealed that a concurrent overnight exposure to ABT-737 (20 μM) and WP1130 resulted in PARP cleavage and cell death, indicating apoptosis induction. In these treated cells, PARP cleavage increased in a dose-dependent fashion under exposure to 3 μM, 4 μM, and 5 μM WP1130 when co-treated with ABT-737 (Figure 5c). Flow cytometric analysis of H1299 cells confirmed an increased sensitization to ABT-737 under WP1130 exposure by revealing that the percentage of apoptotic cells was significantly higher when cells were treated with both agents compared with individual treatments (Figure 5d).

## Discussion

Our present data clearly demonstrate that the overexpression of Mcl-1 in coordination with Bcl-2/Bcl-xL expression protects cancer cells from apoptosis. Mitochondria are the main ATP producers in cells and are therefore essential for all cellular processes. Furthermore, mitochondria play a pivotal role in life or death decisions in the cell by regulating the apoptosis pathway. The release of cytochrome C from mitochondria leading to the activation of caspases is a hallmark of the apoptotic response. Concomitantly, resistance to apoptosis can arise from a reduction in mitochondrial outer membrane permeabilization. Akt kinase, autophagy, and elevated Bcl-xL and Mcl-1 can cooperate to protect tumor cells against chemotherapy-induced apoptosis by maintaining mitochondrial stability [24,25]. The NIH Developmental Therapeutics Program has determined that Bcl-xL may play a unique role in the general resistance of cancer cells to cytotoxic agents by showing that a variety of cancer cell lines that demonstrate resistance to 70,000 cytotoxic agents are characterized by high Bcl-xL expression [26]. Mcl-1 overexpression has also been reported to contribute to chemoresistance in multiple tumors [15,27] and, notably, has been implicated in the chemoresistance of certain types of malignancies to the first of a new class of Bcl-2-family targeting compounds, ABT-737 [28].

Because of the overexpression and overlapping functions of the Bcl-2 family proteins, it will be important to develop an inhibitor of both Bcl-2/xL and Mcl-1. It has been shown previously that either Mcl-1 downregulation or NOXA overexpression, an Mcl-1 specific BH3-only protein, strongly sensitizes melanoma cells to ABT-737 in vitro [7]. Hence, developing BH3 mimetics could be a feasible approach to inhibit Mcl-1 function. Unfortunately, none of the BH3 mimetics under current development are potent and specific Mcl-1 antagonists [29]. Indeed, many “pan- Bcl2 inhibitors” suffer from a lack of specificity or are simply too weak to compete with native high-affinity BH3-only proteins for pro-survival BH3 binding pockets. Further, such pan-Bcl2 family protein inhibitors might well damage normal tissues. Hence, BH3 mimetics specific for single pro-survival targets could have greater clinical utility [30]. Pertinently, GDC-0199, a novel BH3 mimetic developed by Abbott and Genentech that is specific for Bcl-2, and which is now entering clinical trials for lymphoid malignancies, should avoid the dose-limiting thrombocytopenia associated with the navitoclax [31]. For these reasons, designing an Mcl-1 specific inhibitor or searching for alternative targets for Mcl-1 antagonism has become “popular”.

Our current research suggests that USP9X regulates Mcl-1 expression in cancer cells. Deubiquitinases have been demonstrated previously to antagonize specific oncogenic and tumor suppressive E3-ligases and are viewed as emerging targets for cancer therapeutics [32]. USP9X can now be added to this list due to its role in deubiquitination and in stabilizing Mcl-1, a bona fide oncogene. In our current analyses, USP9X expression was found to be strongly associated with Mcl-1 expression in the human cancer tissue samples we tested. Recent reports have suggested also that USP9X enhances Mcl-1 stability by preventing its proteasomal destruction through de-ubiquitination [33]. The balance between ubiquitination and deubiquitination determines Mcl-1 stability and expression. Ubiquitination of Mcl-1 promotes USP9X-Mcl-1 binding leading to Mcl-1 deubiquitination and disassociation of these two proteins. Hence, and as shown from our current data, increasing Mcl-1 ubiquitination via PS341 promotes the association of USP9X with Mcl-1. Since Mcl-1 proteins are constantly ubiquitinated, their association with USP9X appears to be a “steady-state” condition. This activity and upregulation of USP9X as well as Mcl-1 have been associated with a poor prognosis and with chemoresistance in a number of cancers. To determine the impact of USP9X inhibition on cancer cell survival in our present experiments, we used its inhibitor WP1130 and found that the treated cells showed Mcl-1 downregulation which increased their sensitivity to ABT-737 as well as to other chemotherapeutic agents. In light of the importance of USP9X in the control of Mcl-1 levels, compounds such as WP1130 or other more specific inhibitors may be useful in overcoming the apoptotic resistance associated with USP9X activity and Mcl-1 protection. WP1130 may therefore have utility as a chemosensitizer in a combinational chemotherapy regimen as it can inhibit several USPs including USP9X, USP5, USP14, and UCH37, which are known to regulate cell survival, protein stability, and 26S proteasomal function [34]. Furthermore, USP9X is a deubiquitinase that targets multiple proteins involved in cell growth and survival [35,36]. Hence, the design of a specific inhibitor that targets the USP9X and Mcl-1 interaction could also be a viable and possibly even a better approach to reducing the impact of chemoresistance in different tumors.

## Conclusions

Our current analyses demonstrate in principle that the expression of USP9X, Mcl-1 and Bcl-xL contributes to chemoresistance in cancer cells. Promoting Mcl-1 ubiquitination and degradation using USP9X inhibitor sensitizes tumor cells to various chemotherapies including Bcl-2/Bcl-xL inhibitors.

## Abbreviations

USP9X: Ubiquitin specific peptidase 9, X-linked; 5-FU: Fluorouracil; CHX: Cycloheximide; siRNA: Small interfering RNA.

## Competing interests

The authors declare that they have no competing interests.

## Authors' contributions

CP, DJ and XC designed and performed the research, analyzed the data and drafted the manuscript; SF, WJ, JLY, AR, RT, PR, MKNR and WRS performed some of the research and analyzed the data. All of the authors read and approved the final manuscript.

## Pre-publication history

The pre-publication history for this paper can be accessed here:

http://www.biomedcentral.com/1471-2407/12/541/prepub

## Supplementary Material

Additional file 1 **Figure S1.** A549 cells were exposed to a proteasomal inhibitor PS341 for 2, 4 and 6 h and the cells were then harvested and lysed. Ubiquitylated proteins were immunoprecipitated from these lysates using ubiquitin protein enrichment kit from Pierce. Ubiquitylated Mcl-1 signals were then detected by western blotting. Experiments were repeated twice. Click here for file
