# Supplementary figures and images for: The downregulation of Mcl-1 via USP9X inhibition sensitizes solid tumors to Bcl-xl inhibition
Source: BMC Cancer. 2012 Nov 21;12:541. doi: 10.1186/1471-2407-12-541 (PMC3543233; doi:10.1186/1471-2407-12-541)

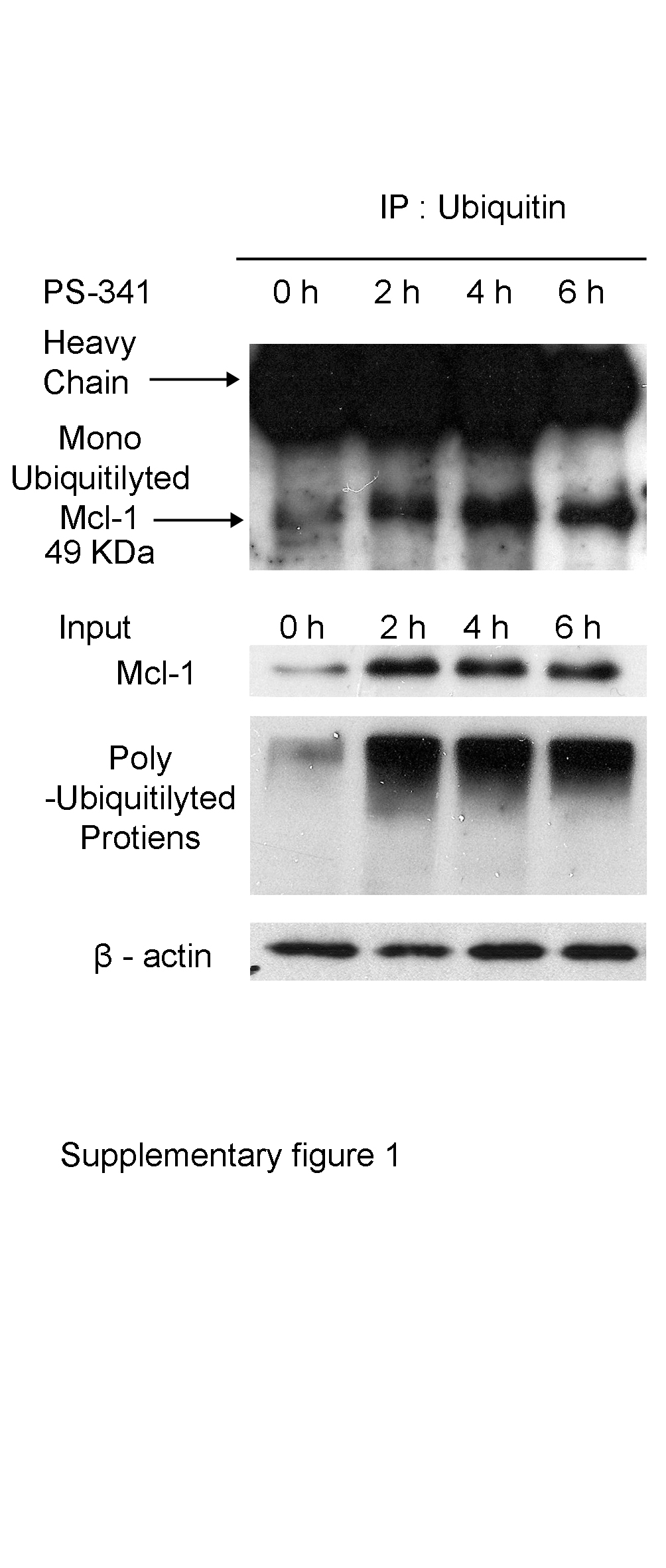

Supplement: Additional file 1 — Figure S1. A549 cells were exposed to a proteasomal inhibitor PS341 for 2, 4 and 6 h and the cells were then harvested and lysed. Ubiquitylated proteins were immunoprecipitated from these lysates using ubiquitin protein enrichment kit from Pierce. Ubiquitylated Mcl-1 signals were then detected by western blotting. Experiments were repeated twice. [file 1471-2407-12-541-S1.jpeg]
